# Supplementary material for: An evaluation to determine if reading the mind in the eyes scores can be improved through training
Source: PLoS One. 2022 Apr 28;17(4):e0267579. doi: 10.1371/journal.pone.0267579 (PMC9049333; doi:10.1371/journal.pone.0267579)
Supplement: Attachment — Submitted filename: Response to Reviewers.docx [file pone.0267579.s001.docx]

**Author Responses to Address Reviewer Comments**

**Paper Title:** An Evaluation to Determine if Reading the Mind in the Eyes Scores Can Be Improved Through Training

**Submitted to:** PLOS One

**Manuscript Number:** PONE-D-21-26174

We would like to thank the reviewers for their feedback and helpful comments to improve this work. They were extremely helpful and well targeted towards improving the paper. We assigned numbers to the comments so we could refer any of them back when there is similar comments from different reviewers.

# Editor

[E.1] Please include your full ethics statement in the ‘Methods’ section of your manuscript file. In your statement, please include the full name of the IRB or ethics committee who approved or waived your study, as well as whether or not you obtained informed written or verbal consent. If consent was waived for your study, please include this information in your statement as well.

[Author Response] We added the following statement: "This study was approved as exempt by the Institutional Review Board of Iowa State University (#18-075). Electronic informed consent was obtained from all participants.”

[E.2] Please ensure that you refer to Figure 1 and 3 in your text as, if accepted, production will need this reference to link the reader to the figure

[Author Response] The citation of Figure 1 has been added to the text. Figure 3 was deleted to address later review comments.

[E.3] Please ensure that your manuscript meets PLOS ONE's style requirements, including those for file naming. The PLOS ONE style templates can be found at https://journals.plos.org/plosone/s/file?id=wjVg/PLOSOne_formatting_sample_main_body.pdf and https://journals.plos.org/plosone/s/file?id=ba62/PLOSOne_formatting_sample_title_authors_affiliations.pdf

[Author Response] All style requirements have been implemented in the manuscript.

[E.4] Should your manuscript be accepted for publication, we will hold it until you provide the relevant accession numbers or DOIs necessary to access your data. If you wish to make changes to your Data Availability statement, please describe these changes in your cover letter and we will update your Data Availability statement to reflect the information you provide.

[Author Response] We have an Open Science Framework repository for this study’s data linked here: https://osf.io/bd4rp/

# Reviewer #1

[R1.1] The references in the Introduction section are outdated. A lot of work has been done in the last decades to understand the steps of ToM development. Please add more recent references, and more nuance in some assertions (for example page 6: “Due to the proposed reliance on instinct in the process, we believe ToM is best attributed to System 1 thinking, theorized by Kahneman (2011).).” See also major point number 5 on a related matter.

[Author Response] More recent references have been added to the introduction in the areas of training and the potential for training to be useful in creating better Human-AI collaboration. The assertion on page 6 has been removed as it was unnecessary when introducing ToM. Major point 5 is concerned with a potentially missing reference, Authors, 2018. This author names have been redacted for blind review, but the paper has been published.

[R1.2] Page 6: I don’t see how referencing Kahneman’s framework is useful to understand the experiment or the results. Same for believers related data page 7. Same for mood related data page 7.

[Author Response] Referencing Kahneman's framework is not necessary when introducing ToM, the experiment, or the results. Therefore, the assertion on page 6 has been removed. As part of the effort to the streamline the focus of the manuscript on the training, we have edited the manuscript to no longer include mentions of the research on the impact of belief in a god and mood, and other demographic variables, on the ability to engage in mindreading, or reading the mind in the eyes.

[R1.3] 3. Page 11: “Analogous to the concept of diminishing returns”. Please provide a definition and references to back up the concept of “diminishing returns”.

[Author Response] The connection to "diminishing returns" was unnecessary in explaining the hypothesis. Therefore, the statement has been removed

[R1.4] 4. Page 12: “Because mental state interpretation is quick, automatic, and universal”. Whether mental state understanding is automatic, universal or quick is the subject of entire research programs. I don’t think these questions are elucidated to this day. Please provide references to back up this assertion, and develop a theoretical argument to justify it, or either drop or adjust the assertion.

[Author Response] The assertion has been removed from the paper as it was unnecessary in explaining the inclusion of H4.

[R1.5] 5. Page 14: “while more detail can be found in Authors (2018).” Please refrain from citing unpublished data. I couldn’t find the referenced work on the internet.

[Author Response] This is a published paper, and was referenced as "Authors" for the blind review. It was published in the proceedings of HFES 2018

[R1.6] Page 19: “One-way ANOVAs were used to inspect the simple main effects of training condition,”… I don’t see the point of this analysis. The previous ANOVA on change scores, with Training Type as factor, already showed a main effect of High Vs Low Scorers. Why then use three different ANOVAs for each type of training to just show again this effect for each training? If the authors want to explore the marginal effect, they should instead run two additional ANOVAs: one for the High Scorers with Training Type as factor, and the same for the Low Scorers.

[Author Response] Reviewer 1's suggestion to reduce the complexity of the results by running two separate ANOVAs to replace the three one-way ANOVAs is a clear improvement, and as such, we have redone our analysis to explore the marginal effects of score on training type and updated the discussion and results as necessary.

[R1.7] Page 19: “As predicted, the effect of previous experience with the RMET on Pre-RMET score was significant, t(426) = -2.41, p = .008. (Figure 4a). »… I don’t see how this was predicted in any of the hypotheses listed at the end of the Introduction. Please add the corresponding hypothesis in the Introduction and justify it, or correct this section. What was predicted is less progression between pre and post training for participants with previous exposure to the RMET, not lower scores at pre-training (see H3a). Please also edit the Discussion section, page 24. “In support of H3A, participants with previous experience taking the RMET performed significantly worse than participants who had no previous experience with the RMET.”

[Author Response] Reviewer 1 is correct that the results discussed on page 19 and visualized in Figure 4a is not related to H3A. This text was included mistakenly, and was meant to, instead, give more context to the findings (similar to the distribution of genders across conditions) and the (new) placement of the other demographic variable discussion. To correct this, as well as address Reviewer 2's comment about paring down the arguments to those which are essential, all of figure 4 has been removed.

[R1.8] The authors say in the Introduction section that empathy could be a mediating factor explaining why women (or hypothetically marginalized groups), could perform better on the RMET. Why not include a measure of empathy in the present experiment? Could the authors discuss this matter?

[Author Response] Reviewer 2 suggested that we reduce the number of hypotheses and restructure the arguments of the introduction. As we did not measure Empathy, we have dropped hypothesis H4 and the related discussion of empathy as a mediating factor.

[R1.9] Conclusion section. This section usually contains a single paragraph outlining the main results of the study. Please summarize.

[Author Response] We have rewritten the Conclusion section to a single paragraph that summarizes the results and suggests future work.

[R1.10] The “Related work” section seems not appropriate for a peer reviewed article. It presents data that are not relevant to the experiment. Please extract the relevant information from this section, explicitly link them to your experiment and hypotheses, and drop irrelevant literature.

[Author Response] The Related Work section has been re-structured to focus on training. Other topics, such as the effect of demographics on RMET score, have been removed.

[R1.11] 11. Since the present design is across subjects, it is possible that the Qualtrics randomization led to potential biases. For example, it is possible that a majority of female participants was in the “Repetitive Training” condition, then leading to the effect of training being confounded with the gender factor. Could the authors provide the repartition of participants and their characteristics in each training category? Could they also discuss this potential bias in the Discussion section?

[Author Response] In the (newly named) Data Integrity section (under Experimental Methods), we have added a Table that contains a description of participant characteristics by condition (Table 1). In addition we have added a statement indicating that there is a relative balance of demographic groups across conditions.

[R1.12] 1. There is a typo in Baron-Cohen’s citations. There should be no space after “Baron-“. Please correct.

[Author Response] Thank you for catching this. It has been corrected.

[R1.13] . I don’t see the use of Table 1… Rather, a Table showing the characteristics of participants in each training group would be more useful.

[Author Response] Thank you for this suggestion. We have added a table (Table 1) that gives counts by gender, race/ethnicity, native English speaker status, US-native status, and age for each condition.

[R1.14] 3. Please move the Limitations and Assumptions section to the end of the paper.

[Author Response] The Limitations and Assumptions section has been moved to the end of the Discussion section, just before the (summary) Conclusion.

[R1.15] 4. Page 19: “Figure 2Error! Reference source not found.” Please correct.

[Author Response] The Figure 2 citation has been corrected

[R1.16] 5. Page 23: “, but a marginally significant, weak, and negative association was uncovered between overall RMET Score and overall average time per question”. I think that this part of the sentence refers to high skill participants, but this needs to be explicit. Please correct.

[Author Response] That is correct, the second part of the sentence refers to high skill participants. The sentence has been rewritten to read, "but a marginally significant, weak, and negative association was uncovered between overall RMET Score and overall average time per question (τb = -.11, p = .09) for high-skill participants."

# Reviewer #2

[R2.1] The paper would benefit from a change of the structure. The introduction should be focused on the topic of the study, that is the training, and not on other irrelevant information such as benefits of higher Reading the Mind in the Eyes Ability or studies on theory of mind. I would suggest discussing in a critical way the training interventions reported in the literature. This could help the Authors explain the relevance and novelty of their study, at the moment it is not clear and defined.

[Author Response] We have rewritten the Introduction to focus on the central theme of the paper: can RMET be trained. We have added more discussion on previous work in training. The section on the benefits of higher RMET score has been removed from the literature review, as well as the material on demographics.

[R2.2] The method section needs to be more specific. Several information such the length of the training should be added.

[Author Response] We have added detail to the method section including: ethics statement, the length of the experiment overall, the length of each condition, limitations, and participant demographics by condition.

[R2.3] The procedure section is very important, and I do not think is well presented. This makes difficult the interpretation of the results.

[Author Response] The procedure section has been restructured to better convey at what point in the study participants were split into conditions and the differences among the three conditions.

[R2.4] I do not think that the label “strategic training” is correct, I would suggest using something different that highlights the content of the intervention.

[Author Response] We've changed the condition label to "naturalistic" to reference the trainings' focus on identifying emotions in naturalistic settings (i.e., observing human faces or anthropomorphic faces in more context, including the addition of a mouth or narrative arc).

[R2.5] The limitations of the study should be moved to the end of the discussion section

[Author Response] The limitations were moved to the end of the Discussion section.

[R2.6] the aims should be presented in the introduction

[Author Response] The aims of the study have been added to the last paragraph of the Introduction, and reads, "The aim of this work was to develop and test methods of training individuals to perform better on the RMET."

[R2.7] I would suggest reducing the number of hypotheses.

[Author Response] Upon reviewing our hypotheses, we have removed H4. This information deserves its own fully-fledged study, and it is beyond the scope of our original question about training. Thus, because of the breadth of our study, the demographic variables are no longer included in the hypotheses, only as discussion points. We also have removed H3A, as this hypothesis distracted from the focus of the paper.
