# Supplementary material for: An evaluation to determine if reading the mind in the eyes scores can be improved through training
Source: PLoS One. 2022 Apr 28;17(4):e0267579. doi: 10.1371/journal.pone.0267579 (PMC9049333; doi:10.1371/journal.pone.0267579)
Supplement: Attachment — Submitted filename: Response to Reviewers.docx [file pone.0267579.s002.docx]

**Author Responses to Address Reviewer Comments**

**Paper Title:** An Evaluation to Determine if Reading the Mind in the Eyes Scores Can Be Improved Through Training

**Submitted to:** PLOS One

**Manuscript Number:** PONE-D-21-26174

We would like to thank the reviewers for their feedback and helpful comments to improve this work. They were extremely helpful and well targeted towards improving the paper. We assigned numbers to the comments so we could refer any of them back when there is similar comments from different reviewers.

# Editor

[E.1] Please review your reference list to ensure that it is complete and correct. If you have cited papers that have been retracted, please include the rationale for doing so in the manuscript text, or remove these references and replace them with relevant current references. Any changes to the reference list should be mentioned in the rebuttal letter that accompanies your revised manuscript. If you need to cite a retracted article, indicate the article’s retracted status in the References list and also include a citation and full reference for the retraction notice.

[Author Response] We have reviewed the reference list to ensure it is complete and correct.

# Reviewer #1

[R1.1] The authors have added sentences pertaining to human-human interaction or human to AI interaction. What is the rationale for this? Please provide a rationale, if possible with references, or drop this argument.

[Author Response] In recent years, researchers have been looking to guide the creation of AI agents which better approximate human social intelligence. One of the threads of this research leads to Theory of Mind, which underlies the Reading the Mind in the Eyes Test. The sentences pertaining to human-human and human-AI interaction were added to demonstrate the utility of ToM training. The sentences pertaining to human-AI interaction have been rewritten, and another reference [10] added: "In human-AI interactions, the machine’s ability to attribute mental states to others greatly enhances the quality of the interaction [10]. As such, AI researchers have turned to ToM as a way to provide automations with the ability to process human facial expression data in real time [10]"

[R1.2] I am still not satisfied with the “Related Work Section”. It really hinders the interest of your work as it looks more like a patchwork of data rather than a well-constructed introduction. I never found such section in any published article I ever read. I think it would really be better if the authors just picked the relevant parts of this section to inject them in a more streamlined Introduction Section.

[Author Response] The separate "Related Work" section has been removed. The relevant content from this section has been integrated into the Introduction. Additionally, information that was redundant with the content of the introduction was removed.

[R1.3] I don’t understand the rationale for H4. If the authors really follow the line of reasoning in the experiments by Tracy and Robins (2008), they should hypothesize just the opposite, or maybe that there is no correlation between RT and accuracy... I find the H4 hypothesis very confusing and counterintuitive.

[Author Response] Thank you for drawing this to our attention. We have altered the description of our reasoning to clarify that H4 is meant not to take Tracy and Robins line of reasoning and further it, but to attempt to duplicate or validate their findings under different contexts, to combat the ongoing "replication crisis" within social sciences.

[R1.4] Page 18: “the RMET has demonstrated a strong ability to identify individuals with impaired social intelligence but otherwise normal cognitive intelligence”. This phrasing could be interpreted as offensive for people with Autism Spectrum Disorder. Please use "typical" instead of "normal", or another similar word. Please use another expression than "impaired social intelligence". The neurodiversity movement is all about being considered as different people and not as impaired or incomplete beings. So please, if you mention this idea, think about the people involved and how they will interpret your phrasing.

[Author Response] We have replaced "normal" with "typical" and "impaired social intelligence" with "different social intelligence".

[R1.5] Page 18: “There are a few known limitations of the RMET, etc.” and the two following paragraphs. These paragraphs are interesting but should be integrated in the Introduction section. Usually, in the Methods section, when the literature guides choices regarding the methods, references are briefly provided with a one sentence rationale. If the rationale requires a full paragraph, it should be fully developed in the Introduction, and then briefly reminded in the Methods, where the relevant procedure is described

[Author Response] These paragraphs have been integrated into the Introduction section.

[R1.6] Page 19: “Lastly, The RMET involves the association of facial expressions to high-level emotion words which may be difficult for even native English speakers”. Please add at least one reference to back up what is said here.

[Author Response] This statement has been changed to reflect that taking the RMET in a language outside of one's native language may impact score. Additional references have been added: “Lastly, The RMET involves the association of facial expressions to emotion words which may be difficult for participants whose native language is not English. The difficulty of completing the RMET outside of one’s native language and the utility of offering the test in multiple languages is evidenced by the translation of the test into French [48] and Spanish among others [49].”

[R1.7] Page 23: “One-way ANOVAs were used to inspect the simple marginal effects, etc.” I don't understand why the authors would want to explore marginal effects. Usually one would want to test for differences (at p<.05) and report marginal effects when they are found instead.

[Author Response] This was a hold-over from our last iteration. We thank you for your insight. The statement has been changed to read “Two ANOVAs were used to inspect the marginal effects of training condition at each level of Initial RME ability, with significance evaluated at an alpha level of .025 to adjust for the use of multiple tests.”

*[R1.7] “or it could be evidence of regression to the mean, as explained by Kahneman”. I don't understand this sentence. What is regression to the mean in this context?*

[Author Response] We have added text to explain the statement within the context of our study: “This could indicate that the training is simply not effective for people who are already good at Reading the Mind in the Eyes, or it could be evidence of regression to the mean, a statistical phenomenon showing that high performers regress toward the mean or average while low performers will more typically see performance gains relative to their starting measurement [47]. Regression to the mean, in this context, speaks to the observation that average scores for the top 25% performers were on average one-and-a-half points lower (1.45) in the post-RMET, while the bottom 25% saw net gains of near two points (1.7), bringing each group closer to the average than where they started.”
